# A Rare Presentation of a 12-Year-Old With Systemic Infantile Hyalinosis: A Case Report and Review of the Literature

**DOI:** 10.7759/cureus.66495

**Published:** 2024-08-09

**Authors:** Fatima Alfadhli, Layan Alrehaili, Joud N Bindekhayel, Laila Alzamil, Abdulrahman Alrehaili, Zahera Hussain

**Affiliations:** 1 Pediatric Department, Maternity and Children Hospital, Madinah, SAU; 2 College of Medicine, Al Imam Mohammad Ibn Saud Islamic University, Riyadh, SAU

**Keywords:** diagnostic genetic testing, antxr2 gene, pediatric genetics, rare genetic disease, infantile systemic hyalinosis

## Abstract

This case report presents the clinical manifestation and diagnostic testing of a 12-year-old male diagnosed with systemic infantile hyalinosis (SIH) at the Maternity and Children Hospital in Madinah in 2012. The patient presented with typical SIH symptoms, including painful joint contractures, hyperpigmented knuckles, gingival hypertrophy, subcutaneous nodules, and recurrent infections. Whole exome sequencing (WES) analysis identified a homozygous mutation in the ANTXR2 gene, which is a deletion in exon 13 (c.1074delT; p.A359HfsX50), confirming the diagnosis. Notably, this patient's survival beyond the typical age expectancy of SIH, which is usually within the first few years of life, challenges the usual prognosis associated with this disease. This case emphasizes the importance of early diagnosis through clinical suspicion confirmed by genetic analysis and highlights the variability in disease presentation and prognosis.

## Introduction

Systemic infantile hyalinosis (SIH) is an extremely rare autosomal recessive disorder that primarily affects infants [1]. It is characterized by the abnormal accumulation of hyaline in various tissues and organs throughout the body [2]. SIH is believed to be caused by mutations in the ANTXR2 gene, which is responsible for encoding a protein involved in the breakdown of hyaline [3]. Infants with SIH typically present with a range of symptoms, including extreme joint stiffness, thickened skin, growth retardation, and failure to thrive. Other common features include gastrointestinal symptoms, respiratory difficulties, and neurological abnormalities [4]. The severity of symptoms can vary greatly, but the prognosis is generally poor, with most affected infants succumbing to the disorder within the first year of life [5]. Diagnosing SIH can be challenging due to its rarity and the overlap of symptoms with other disorders. Genetic testing is often necessary to confirm the presence of ANTXR2 gene mutations. Unfortunately, there is currently no cure for SIH, and treatment focuses mainly on supportive care to manage symptoms and improve quality of life. This may involve physical therapy, pain management, respiratory support, and nutritional interventions [6]. In conclusion, systemic infantile hyalinosis is an extremely rare condition with a poor prognosis, as most patients do not survive beyond their third year [7]. However, this case report presents a unique situation, as the patient is 12 years old.

## Case presentation

This is a 12-year-old male, a product of a full-term normal spontaneous vaginal delivery to parents in a consanguineous marriage, with an uneventful birth history. Since early infancy, he has presented with progressive joint contractures, cutaneous lesions, and recurrent infections. His mother first noticed stiffness in his upper and lower extremities and developmental delays around four months of age. At six months, he experienced three episodes of abnormal movements, prompting evaluation at a private clinic, where examination revealed some stiffness and restriction of both knees and elbow joints, raising suspicion of arthrogryposis multiplex congenita. Subsequently, he was referred to Madinah Maternity and Children Hospital for further evaluation at the pediatric specialist clinic.

**Figure 1:**
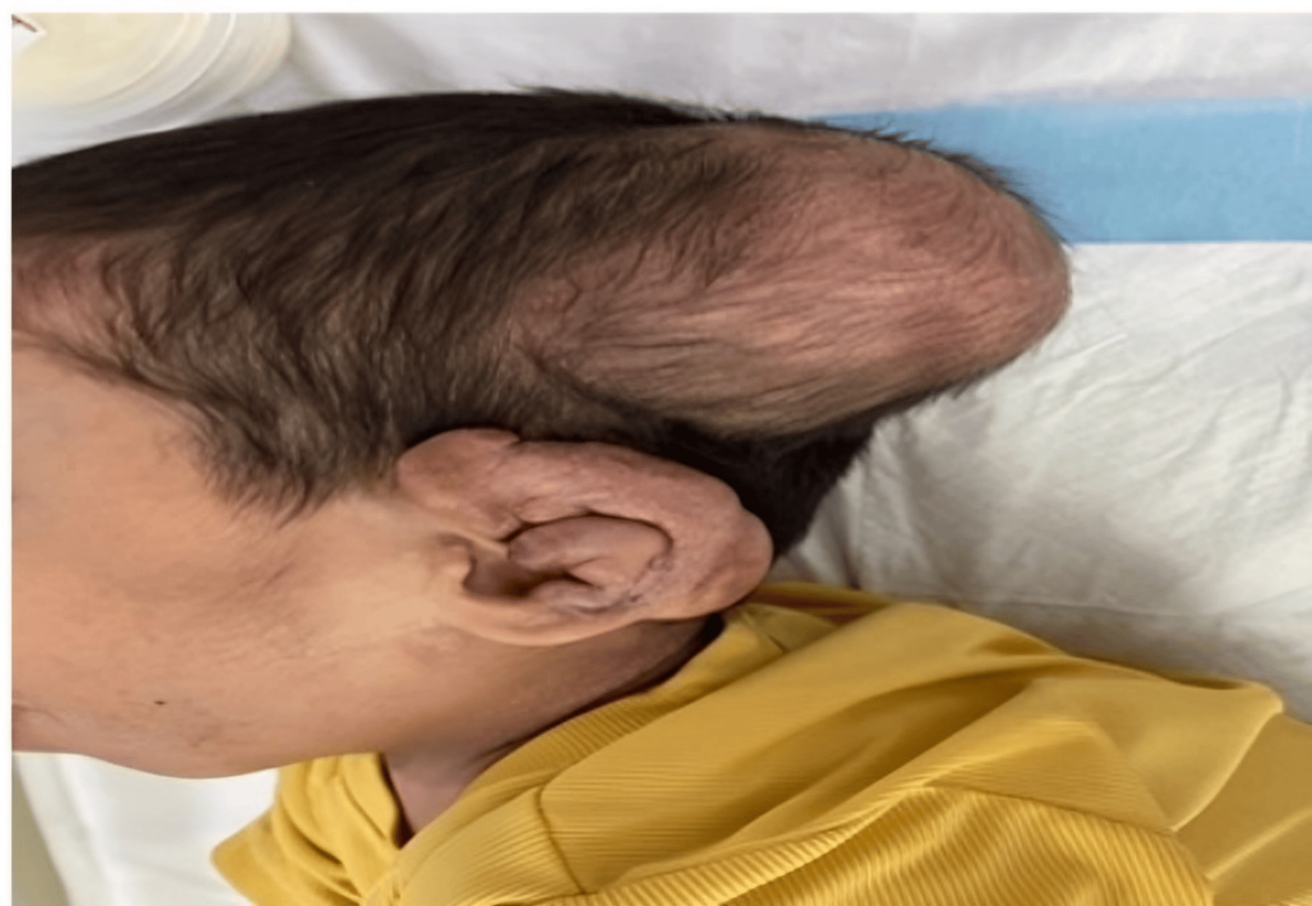
Subcutaneous tumors on the scalp Large subcutaneous tumors on the scalp with diffuse infiltration of the helix of the ear.

**Figure 2:**
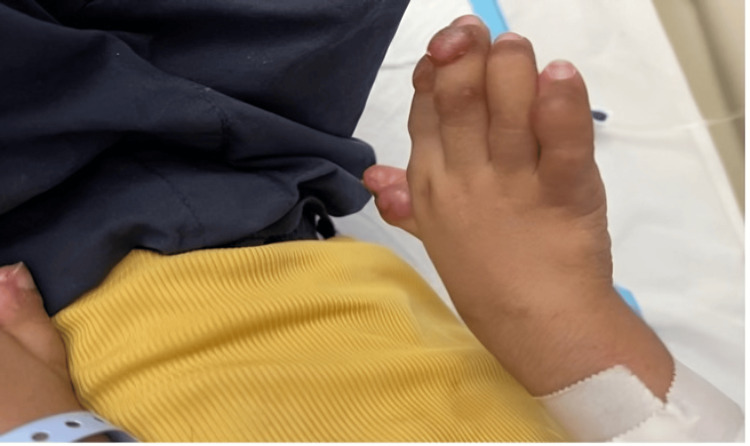
Hyperpigmented nodular lesions Hands are everted with hyperpigmented nodular lesions over the metacarpophalangeal joints with multiple subcutaneous nodules.

**Figure 3:**
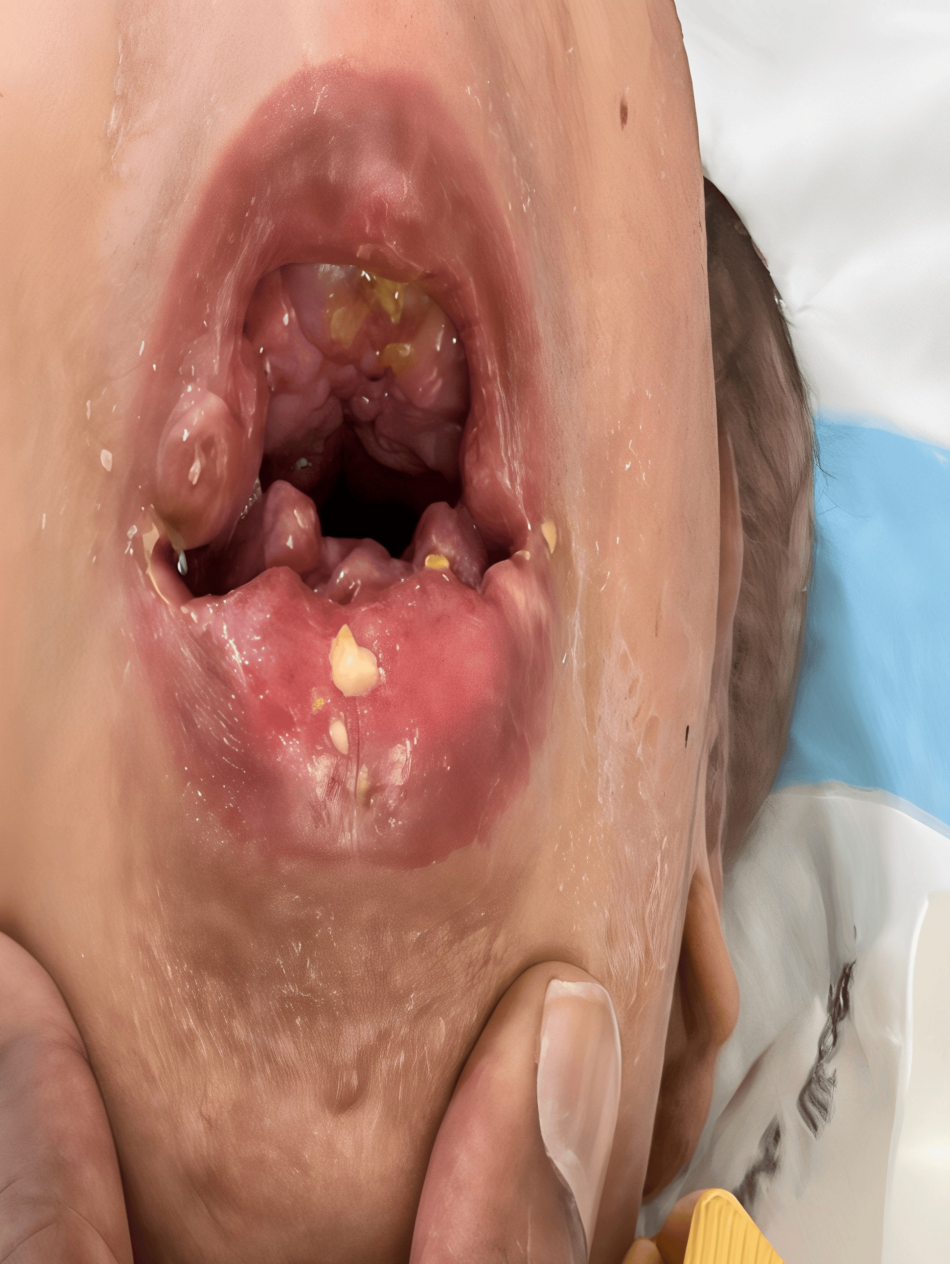
Gingival hypertrophy Marked gingival hypertrophy, which almost covered teeth with an everted lower lip due to a prominent gingival mass.

Initial clinical findings included abnormal posture, hypertonia, muscle wasting, and small nodules around the anus that were continued with the provisional diagnosis of arthrogryposis multiplex congenita, as both diseases present with generalized joint stiffness. Further consultations in medical genetics and neurology were sought at 16 months of age. Neurological assessments revealed restricted joint movements and stiffness. Physical examination highlighted several characteristic features, including large subcutaneous tumors on the scalp, diffuse infiltration of the helix of the ear (Figure 1), hyperpigmented nodular lesions over the metacarpophalangeal joints with multiple subcutaneous nodules (Figure 2), and over both malleoli, massive gingival hypertrophy (Figure 3), and small perianal nodules.

There was no family history of similar presentations, abortions, or stillbirths. Histopathological examination of excised tumors confirmed their benign nature. This clinical profile, along with histopathological findings, raised suspicion of infantile systemic hyalinosis, prompting genetic analysis. At 23 months of age, whole exome sequencing confirmed the diagnosis, as results showed the WES-targeted mutations in the ANTXR2 gene. It revealed a homozygous disease-causing single nucleotide deletion of frameshift mutation, specifically identified as exon 13 (c.1074delT; p.A359HfsX50) of the ANTXR2 gene, characteristic of infantile systemic hyalinosis.

The family was counseled regarding the genetic nature of the disease, which follows an autosomal recessive inheritance pattern. They were advised on preventive genetic measures for future pregnancies and the multidisciplinary approach of supportive management, including pain relief and symptomatic treatments, as no definitive cure exists.

Management involved a multidisciplinary team including physiotherapists, rheumatologists, neurologists, geneticists, infectious disease specialists, and dermatologists. Gentle physiotherapy was initiated post-diagnosis to address limited painful mobility. Symptomatic management was provided for cutaneous lesions. Moreover, recurrent infections were managed properly as indicated; for that reason, they usually play an important role in worsening the prognosis in such patients. Ongoing close monitoring by the multidisciplinary team ensures timely adjustments to his evolving clinical needs and supportive care.

## Discussion

This case stands out due to the patient's remarkable survival well beyond the typical presentation period. SIH is typically recognized in infancy, and the majority of cases do not survive beyond the first few years of life. The fact that our patient has reached 12 years of age emphasizes the variability in the disease course and challenges the notion that SIH is uniformly fatal in early childhood. The patient exhibited several key clinical features typical of SIH, including painful joint movement restrictions, subcutaneous nodules, hyperpigmentation over bony prominence, and marked gingival hypertrophy. Of particular note was the recurrent pattern of infections observed, which is consistent with the immunological deficiencies commonly associated with SIH. These infections further highlight the systemic nature of the disease and the impact of hyaline accumulation on the immune system. Investigating the clinical findings and conducting a thorough analysis of genetic mutations in CMG2/ANTXR2 can enhance our understanding of the disease's pathogenesis. However, the diagnosis of the disease is based on clinical findings and molecular diagnosis, as well as other investigations. Notably, skin biopsy may reveal the accumulation of hyaline material in the dermis in some cases [1,2,4-7], whereas others, like ours, may not demonstrate this characteristic. Additionally, an intestinal biopsy may indicate villous atrophy and lymphangiectasia, while skeletal X-rays can show osteopenia, periosteal reaction, and lucent lesions, as in some cases [4,6,7]. In our patient and similar reported cases [5,6], the diagnosis of SIH was confirmed through genetic testing for the ANTXR2 gene. Furthermore, the presence of abundant homogenous amorphous eosinophilic material with minimal cellular content in the dermis served as a crucial diagnostic feature in most cases described in the literature [1,2,5-7]. Although molecular analysis of the ANTXR2 gene can serve as the primary confirmatory investigation for diagnosis, these mutations disrupt the normal function of ANTXR2, leading to the accumulation of hyaline material in various tissues such as the skin, joints, and internal organs. Nonetheless, the exact mechanism through which hyaline accumulates and its role in the progression of the disease remains poorly understood, highlighting the need for further research in this area. SIH is an exceedingly rare autosomal recessive genetic disorder characterized by a wide range of severe symptoms that typically manifest in infancy. It falls under the broader category of connective tissue disorders. This condition is caused by mutations in the ANTXR2 gene, which leads to the abnormal accumulation of hyaline in various tissues like the skin, musculoskeletal system, gastrointestinal tract, and multiple organ systems, causing systemic damage. This accumulation may manifest as thickened skin, joint contractures, severe diarrhea, growth retardation, and a host of other complications. Affected infants often face significant pain and distress, and without appropriate intervention, the prognosis is grim, with most not surviving beyond a few years of age. Treatment options for SIH are limited, primarily focused on managing the symptoms and improving the quality of life. Supportive care, including physical therapy and nutrition management, can alleviate some of the pain and discomfort associated with the condition. While research into potential therapies continues, the rarity of this disorder poses significant challenges for the development of targeted treatments. Prevention of SIH is possible through genetic counseling for carriers of the ANTXR2 mutation to minimize the risk of passing on this devastating condition to their children. Furthermore, it highlights the need for continued research into understanding the underlying mechanisms and potential therapeutic interventions for such a rare genetic disorder.

## Conclusions

This case challenges the typical prognosis of infantile systemic hyalinosis (ISH) by highlighting the patient's survival well beyond infancy. It underscores the importance of early clinical suspicion and recognition of the disease, followed by genetic mutational analysis confirmation, and early involvement of multidisciplinary care in managing rare genetic disorders like SIH. Further research is needed to understand SIH's mechanisms fully. This case emphasizes the necessity for ongoing monitoring and supportive care by multidisciplinary teams to optimize outcomes for patients with rare genetic disorders.
